# Comparative efficacy of once-daily versus twice-daily doxycycline regimens in dogs naturally infected with *Ehrlichia canis*: A randomized clinical trial

**DOI:** 10.1016/j.vas.2026.100661

**Published:** 2026-04-16

**Authors:** Mookmanee Taechikantaphat, Areerat Kongchareon, Pruksa Julapanthong, Ladawan Sariya, Walasinee Sakcamduang

**Affiliations:** a Department of Clinical Sciences and Public Health, Faculty of Veterinary Science, Mahidol University, Nakhon Pathom 73170, Thailand; b Prasu Arthorn Veterinary Teaching Hospital, Faculty of Veterinary Science, Mahidol University, Nakhon Pathom 73170, Thailand; c The Monitoring and Surveillance Center for Zoonotic Diseases in Wildlife and Exotic Animals, Faculty of Veterinary Science, Mahidol University, Nakhon Pathom 73170, Thailand

**Keywords:** Canine, *E. canis*, Platelet recovery, Tick-borne disease, Treat-to-target

## Abstract

This prospective, randomized clinical trial compared the therapeutic efficacy and rate of platelet recovery of doxycycline 10 mg/kg once daily (SID) (Group A, n=17) versus 5 mg/kg twice daily (BID) (Group B, n=12) in 29 dogs naturally infected with *Ehrlichia canis*. Using a "treat-to-target" protocol that mandated both molecular clearance and platelet normalization prior to cessation, results showed that while both regimens achieved 100% molecular clearance, the BID regimen demonstrated significantly more rapid hematological recovery (P < 0.05 at Days 7 and 14) and required a significantly shorter median treatment duration (28.5 days vs. 39.0 days; P = 0.015). Notably, delayed molecular clearance (>28 days) was observed exclusively in the SID group, and post-treatment recrudescence was detected in two dogs at Day 98. We conclude that the 5 mg/kg BID protocol offers superior pharmacodynamic performance, and a "treat-to-target" strategy involving long-term monitoring is recommended over a fixed 28-day course to ensure complete clinical and parasitological resolution.

## Highlights

• BID doxycycline significantly reduces treatment duration.
• Divided doxycycline dosing accelerates early platelet recovery.
• Standard 28-day doxycycline therapy may fail to completely clear *E. canis* DNA.
• A treat-to-target doxycycline protocol ensures complete molecular cure.

## 1 Introduction

Canine monocytic ehrlichiosis (CME), caused by the intracellular rickettsial tick-borne organism *Ehrlichia canis* (*E. canis*), a gram-negative obligate intracellular bacteria of the genus *Ehrlichia*, family Anaplasmataceae (Aziz et al., 2023; Harrus & Waner, 2011). It remains a tick-borne disease of global veterinary importance, particularly in tropical and subtropical regions, including Thailand (Chamsai et al., 2024; Little, 2010; Osathanon et al., 2025; Waner & Harrus, 2013). Transmitted by the brown dog tick, *Rhipicephalus sanguineus*, the disease manifests in acute, subclinical, and chronic phases, often characterized by thrombocytopenia, fever, bleeding diathesis, and hyperglobulinemia (Boontuboon et al., 2026; Cohn, 2003; Harrus et al., 2016; Mylonakis et al., 2019; Mylonakis et al., 2006; Neer et al., 2002; Waner & Harrus, 2013). Co-infections with other hemopathogens such as *Babesia* spp. and *Hepatozoon* spp. are common in endemic areas and can complicate clinical presentation and therapeutic outcomes (Chamsai et al., 2024; Kledmanee et al., 2009; Osathanon et al., 2025; Otranto et al., 2010).

Doxycycline is currently regarded as the first-line therapy for the treatment of CME (Mylonakis et al., 2019; Neer et al., 2002). However, despite its widespread use, the optimal dosage regimen and duration of therapy remain subjects of ongoing debate in veterinary literature (Mylonakis et al., 2019). The 2002 American College of Veterinary Internal Medicine (ACVIM) consensus statement recommends a standard protocol of 10 mg/kg orally once daily (SID) for 28 days, regardless of the disease phase (Neer et al., 2002). Nevertheless, empirical evidence suggests that this standardized approach may not guarantee pathogen elimination in all cases. In practice, veterinarians frequently encounter cases of unsuccessful or incomplete treatment, characterized by persistent subclinical infections or clinical recurrence after therapy cessation (Fourie et al., 2015; Harrus et al., 1998). While the 10 mg/kg SID regimen has demonstrated efficacy in some studies (Harrus et al., 2004; Schaefer et al., 2008), conflicting reports indicate that it may fail to achieve molecular clearance in a subset of naturally and experimentally infected dogs. Notably, several studies have documented the persistence of *E. canis* DNA despite extended treatment durations of 4 to 6 weeks at this dosage, even when clinical and hematological recovery was achieved (Fourie et al., 2015; Harrus et al., 1998; McClure et al., 2010).

Conversely, alternative regimens utilizing 5 mg/kg administered every 12 hours (BID) have shown promise in effectively eliminating infection in both acute and subclinical phases within 2 to 4 weeks (Breitschwerdt et al., 1998; Neer et al., 1999; Shropshire et al., 2018). This creates a critical disconnect between apparent clinical resolution and true parasitological clearance. Because *E. canis* sequesters within mononuclear phagocytes in tissue reservoirs (e.g., spleen and bone marrow), hematological parameters often normalize rapidly as systemic inflammation subsides, masking the ongoing infection. Given the bacteriostatic nature of doxycycline, premature cessation of therapy based solely on improved blood counts may fail to eradicate these reservoirs, ultimately leading to persistent subclinical infection or subsequent recrudescence (Harrus et al., 1998; McClure et al., 2010). Consequently, there is a pressing need to directly compare the efficacy and hematological recovery between the standard SID and the divided BID doxycycline regimens in a controlled clinical setting. Therefore, the objective of this prospective, randomized clinical trial was to compare the therapeutic efficacy, rate of platelet recovery, and total treatment duration between the 10 mg/kg SID and 5 mg/kg BID doxycycline regimens in dogs naturally infected with *E. canis*.

## 2 Materials and methods

### 2.1 Study design, setting, and ethical considerations

This prospective, randomized clinical trial was conducted at Prasu Arthorn Veterinary Teaching Hospital, Faculty of Veterinary Science, Mahidol University, between December 2016 and December 2018. The study protocol was reviewed and approved by the Mahidol University-Institute Animal Care and Use Committee (MU-IACUC) of the Faculty of Veterinary Science (Approval No. MUVS-2016-09-40). The study population consisted of adult dogs of both sexes, including purebreds and mixed breeds. All dogs were privately owned patients living in domestic environments, housed either indoors or outdoors, with or without other animals. Feeding and husbandry were maintained according to the owners' normal practices. The study protocol was explained to the owners, and informed consent was obtained prior to enrolment.

### 2.2 Inclusion and exclusion criteria

Dogs suspected of canine monocytic ehrlichiosis (CME) were screened for eligibility based on specific serological, clinical, and hematological criteria.

#### 2.2.1 Inclusion criteria

To be eligible for enrolment, dogs were required to test positive for *E. canis* antibodies using a commercial rapid test kit (Witness® *Ehrlichia,* Zoetis (Thailand) Ltd, Bangkok, Thailand) and concomitantly present with at least one of the following clinical signs or laboratory abnormalities: depression or lethargy, anorexia, tick infestation, fever (>102.9°F), weight loss, bleeding tendency, inflammatory ocular disease, hematocrit <35%, leukopenia (<6,000 cells/µL), or thrombocytopenia (<150 × 10^3^/µL).

#### 2.2.2 Exclusion criteria

Dogs were excluded from the study if they met any of the following conditions: a known history of trauma, administration of any anti-*Ehrlichia* treatment within 30 days prior to examination, confirmed co-infection with *Babesia* spp. or *Hepatozoon* spp. by multiplex polymerase chain reaction (PCR), or a negative PCR result for *E. canis*, regardless of their initial serological status.

### 2.3 Sample collection and baseline evaluation

At enrolment (Day 0), a 5-mL blood sample was aseptically collected from the cephalic vein or the lateral saphenous vein of each dog using a 22-gauge needle and a 5-mL syringe. The collected blood was immediately aliquoted into three fractions for comprehensive analysis: the first fraction was transferred into a K3-EDTA tube for complete blood count (CBC) analysis using an Animal Blood Counter ABX Micros ESV60 (HORIBA ABX Diagnostic (Thailand) Ltd., Bangkok, Thailand) (Riengvirodkij et al., 2025; Sakcamduang et al., 2025) and serological *E.canis* screening (Witness® *Ehrlichia*); a second fraction was transferred into a separate K3-EDTA tube dedicated for molecular confirmation via multiplex PCR (Chamsai et al., 2024; Osathanon et al., 2025); and the remaining whole blood was transferred into a plain tube and centrifuged at 3000 rpm for 10 minutes at 4°C to separate serum. The harvested serum was analyzed for biochemical profiles, using a Sapphire 400 Auto-Chemistry Analyzer (D.A.P. Siam Group Ltd., Bangkok, Thailand) (Riengvirodkij et al., 2025; Sakcamduang et al., 2025).

### 2.4 Randomization and treatment protocol

Initially, a total of 40 dogs meeting the inclusion criteria were enrolled. Randomization was performed using a systematic allocation method based on the chronological order of enrolment following informed owner consent. Dogs were assigned sequential study identification numbers; those with odd numbers were allocated to Group A, while those with even numbers were allocated to Group B.

Treatment was initiated immediately following randomization based on rapid serological testing. However, final retention in the data analysis remained contingent upon confirmatory multiplex PCR results (approximately 3–5 days post-enrollment), with non-qualifying dogs subsequently excluded as per the predefined criteria.

Enrolled dogs were assigned to one of two therapeutic protocols: Group A received doxycycline monohydrate (Vibravet®, Zoetis (Thailand) Ltd., Bangkok, Thailand) at 10 mg/kg orally once daily (SID), while Group B received the same medication at 5 mg/kg orally twice daily (BID). Concomitantly, all dogs were maintained on a strict tick control program using topical selamectin (Revolution®, Zoetis (Thailand) Ltd., Bangkok, Thailand) throughout the study period. The duration of doxycycline administration was individualized, with a mandatory minimum course of 28 days. Discontinuation of therapy was strictly contingent upon the concurrent fulfillment of four composite criteria: (1) molecular clearance evidenced by a negative PCR result for *E. canis* DNA, (2) quantitative platelet recovery (platelet count > 200 × 10^3^/µL), (3) qualitative platelet recovery confirmed by adequate estimation on blood smear, and (4) complete clinical resolution with normalization of hematological and biochemical profiles. Dogs failing to meet these criteria continued treatment and underwent re-evaluation at scheduled intervals until all conditions were satisfied or up to a study duration of 98 days.

### 2.5 Follow-up schedule and monitoring

Following the initial enrollment (Day 0), dogs were subjected to a standardized follow-up protocol with scheduled re-examinations on Days 7, 14, 28, 42, 70, and 98. At each visit, a comprehensive assessment was performed, comprising a physical examination, complete blood count (CBC), and serum biochemistry profiling (total protein, albumin, globulin, ALT, ALP, BUN, and creatinine). To monitor therapeutic efficacy and confirm infection clearance, molecular diagnostics via multiplex PCR for *E. canis* were specifically conducted on Days 28, 42, 70, and 98.

### 2.6 Concomitant management and animal welfare

Throughout the study period, dogs were maintained in their customary home environments with routine husbandry to minimize stress. While standard veterinary care was permitted for minor concurrent conditions, prioritizing animal health and welfare remained paramount. If a dog developed an intercurrent systemic illness requiring medications that could potentially interfere with the *E. canis* treatment protocol, life-saving interventions were prioritized as dictated by good veterinary practice. Such interventions were strictly documented, and the retention of these animals in the final efficacy analysis was subsequently reviewed. The primary therapeutic outcomes evaluated were molecular clearance and clinical/hematological recovery, as defined by the cessation criteria in Section 2.4.

### 2.7 Statistical analysis

Statistical analyses were performed using commercially available software (SPSS version 28.0 for Windows, Chicago, IL, USA). Data normality was evaluated using the Shapiro-Wilk test. Given the relatively small sample size and non-normal distribution of several variables, continuous data are presented as median and interquartile range (IQR), while categorical data are expressed as frequencies and percentages. All statistical tests were two-tailed, and a p-value of less than 0.05 was considered statistically significant.

To ensure clarity and directly link statistical methods to their corresponding outcome variables, the analyses were categorized as follows:

*Baseline characteristics:* Demographic and initial clinical variables between Group A and Group B at Day 0 were compared using the Mann-Whitney U test for continuous variables and the Chi-square test or Fisher’s exact test for categorical variables.

*Time-to-event endpoints:* The time to molecular clearance, time to platelet recovery, and total duration of treatment were estimated using the Kaplan-Meier method. Differences between the survival curves of the two treatment groups were assessed using the Log-rank (Mantel-Cox) test.

*Longitudinal recovery*: To evaluate therapeutic efficacy trends during the initial treatment phase, longitudinal changes in clinical and hematological parameters across four specific time points (Days 0, 7, 14, and 28) were analyzed using the Friedman test. Post-hoc pairwise comparisons to identify significant improvements relative to baseline were performed using the Wilcoxon signed-rank test.

*Long-term stability:* To assess the durability of the therapeutic response, a separate analysis compared parameters across three distinct milestones: baseline, the end of treatment, and the post-treatment follow-up phase. The Friedman test was employed to detect overall differences, followed by Wilcoxon signed-rank tests for pairwise comparisons to evaluate sustained efficacy and confirm the absence of significant regression during the drug-free period.

## 3 Results

### 3.1 Study population and enrollment

A total of 40 dogs presenting with clinical signs suspected of CME were initially enrolled and randomized into two treatment groups (20 dogs per group). Following the screening process, 11 dogs were excluded from the study. In the group assigned to receive doxycycline 10 mg/kg SID (Group A), 3 dogs were excluded due to co-infections confirmed by multiplex PCR: two dogs were co-infected with *Hepatozoon* spp., and one dog was co-infected with *Babesia* spp. In the group assigned to receive doxycycline 5 mg/kg BID (Group B), 8 dogs were excluded. Of these, 5 dogs tested negative for *E. canis* by PCR, and 3 dogs were excluded due to co-infections (two with *E. canis, Babesia* spp., and *Hepatozoon* spp.; one with *E. canis*, and *Hepatozoon* spp.).

A total of 29 dogs met all inclusion criteria and were included in the final analysis (Group A, n=17; Group B, n=12). The study population was predominantly composed of mixed-breed dogs (n=7) and Poodles (n=7). Detailed demographic characteristics, including sex distribution, reproductive status, and the complete distribution of breeds for each group, are comprehensively summarized in Table 1.

**Table 1:** Demographic characteristics, clinicopathological findings, and therapeutic outcomes of dogs naturally infected with *Ehrlichia canis* treated with two doxycycline regimens.

Parameters	Group A (10 mg/kg SID) (n=17)	Group B (5 mg/kg BID) (n=12)	P - value
Age (years)	5.41 (4.91, 7.00)	6.46 (5.73, 7.95)	0.18
Sex (Male/Female)	7/10	5/7	1.00
Reproductive status (n)			0.13
Male (entire/neutered)	4/3	5/0	
Female (entire/spayed)	6/4	6/1	
Breeds (n)			NA
Poodles	7	0	
Mixed	3	4	
Shih Tzus	1	3	
Thais	2	0	
Pomeranians	1	2	
Others#	3	3	
Body Weight (kg)	6.2 (3.2, 7.0)	7.8 (4.0, 11.6)	0.20
Body condition score (5)	3.0 (2.5, 3.5)	3.0 (2.5, 4.0)	0.73
History (n)			
History of tick infestation (%)	16 (94.1%)	11 (91.7%)	1.00
Tick control (no/yes-irregular/ yes-regular)	2/5/10	3/8/1	0.02*
Deworming (no/yes-irregular/ yes-regular)	7/2/8	5/5/2	0.06
Rabies vaccine (yes-within 1-3 year/yes-less than 1 year)	2/15	7/5	0.02*
History of blood parasite infection (no/yes)	12/5	8/4	0.06
Attitude (normal/abnormal)	14/3	9/3	0.47
Appetite (normal/decreased appetite & anorexia)	9/8	3/9	0.32
Water intake (normal/abnormal)	16/1	12/0	0.47
Urination (normal/not observed)	17/0	10/2	0.18
Defecation (normal/not observed)	16/1	11/1	0.46
Coughing (no/yes)	15/2	11/1	0.24
Vomiting (no/yes)	16/1	11/1	0.80
Clinical signs at presentation, n (%)			
Tick Infestation at presentation	11 (64.7%)	8 (66.7%)	1.00
Fever (>102.9°F)	8 (47.1%)	3 (25.0%)	0.27
Ocular signs	5 (29.4%)	3 (25.0%)	1.00
Bleeding diathesis	5 (29.4%)	2 (16.7%)	0.66
Depression/lethargy	3 (17.6%)	3 (25.0%)	0.67
Temperature	102.4 (101.0, 103.6)	102.4 (101.7, 102.6)	0.75
Heart rate (beats/min)	120 (117, 120)	100 (100, 115)	0.03*
White blood cell count (/µL)	7200 (6300, 9200)	8600 (5665, 10025)	0.60
Neutrophil (/µL)	5428 (4722, 6355)	4976 (3766, 7482)	0.51
Lymphocyte (/µL)	1116 (762, 2622)	1818 (959, 2902)	0.29
Monocyte (/µL)	162 (96, 279)	290 (170, 434)	0.07
Eosinophil (/µL)	54 (0, 176)	37 (0, 139)	0.89
Band neutrophil (/µL)	0 (0, 0)	0 (0, 0)	0.77
Red blood cell count (10^6^/µL)	4.98 (3.54, 6.17)	5.20 (3.70, 6.00)	0.89
Hemoglobin (g/dL)	12.5 (8.3, 15.6)	11.6 (7.8, 13.3)	0.45
Hematocrit %	32.8 (24.6, 43.5)	35.9 (23.6, 39.1)	0.71
MCV (fL)	70 (67, 72)	68 (66, 71)	0.36
MCH (pg)	24.5 (23.3, 26.1)	22.3 (21.6, 23.6)	0.01*
MCHC (g/dL)	35.3 (33.8, 37.1)	33.0 (32.0, 34.0)	<0.001*
RDW (%)	15.6 (14.7, 17.2)	15.2 (14.9, 19.1)	0.96
Platelets (10^3^/µL)	48.0 (26.5, 111.5)	36.5 (29.0, 81.3)	0.46
Platelet smear (decreased/adequate)	15/2	12/0	0.22
Plasma protein (g/dL)	10.0 (8.3, 11.6)	8.6 (7.8, 9.6)	0.048*
Total protein (g/dL)	8.4 (7.2, 10.6)	6.9 (6.2, 8.6)	0.04*
Albumin (g/dL)	2.5 (1.9, 2.7)	2.4 (2.1, 2.7)	0.52
Globulin (g/dL)	5.9 (4.7, 8.4)	4.4 (4.2, 5.9)	0.05*
A/G ratio	0.39 (0.25, 0.51)	0.50 (0.43, 0.61)	0.04*
ALP (u/L)	125 (52, 176)	134 (72, 235)	0.61
ALT (u/L)	45 (30, 72)	68 (36, 120)	0.17
BUN (mg/dL)	23 (12, 34)	14 (12, 26)	0.19
Creatinine (mg/dL)	1.0 (0.8, 1.4)	1.0 (0.8, 1.1)	0.25
Dose of doxycycline (mg/kg/day)	11.72 (10.71, 13.39)	12.50 (10.79, 13.15)	0.76
Duration of doxycycline treatment	39.0 (30.0, 57.5)	28.5 (28.0, 37.8)	0.01*
Time to first PCR negative result (days)	28.0 (26.0, 30.5)	28.0 (27.2, 28.8)	0.45
Time to platelet counts > 200 × 10^3^/µL (days)	29 (13.5, 54.5)	13.5 (9.5, 34.0)	0.10
Time to adequate platelet estimation from blood smear (days)	25.0 (9.5, 39.0)	11.0 (7.5, 14.5)	0.07

Data are presented as median (interquartile range, IQR) for continuous variables and frequency (percentage) for categorical variables.

P-value derived from Mann-Whitney U test continuous variables and Fisher’s exact test for categorical variabless.

Abbreviations: SID, once daily; BID, twice daily; MCV: mean corpuscular volume; MCH: mean corpuscular hemoglobin; MCHC: mean corpuscular hemoglobin concentration; RDW: red cell distribution width; A/G ratio, albumin to globulin ratio; ALP: alkaline phosphatase; ALT: alanine transferase; BUN: blood urea nitrogen.

⁎ Statistically significant difference between groups (P < 0.05).

# Other breeds in Group A included French Bulldog (n=1), Golden Retriever (n=1), and Chihuahua (n=1). Other breeds in Group B included Bangkaew (n=1), Chihuahua (n=1), and St. Bernard (n=1).

Baseline demographic data, historical findings, and clinical characteristics are summarized in Table 1. No statistically significant differences were observed between the two groups regarding age, body weight, body condition score (BCS), or sex distribution (P > 0.05). At presentation, the most prevalent clinical signs included tick infestation (65.5%), fever (37.9%), ocular abnormalities (27.6%), and bleeding diathesis (24.1%), with no significant differences in prevalence between the groups. Regarding clinical signs obtained from physical examination, a statistically significant difference was observed in heart rate; Group A had a higher median heart rate compared to Group B (P = 0.03, Table 1), although values in both groups remained within physiological limits. Importantly, this disparity was transient, as no significant differences in heart rate were observed in any subsequent visits. Biochemically, significant disparities were also noted at baseline; Group A exhibited higher median total protein and globulin levels compared to Group B (P = 0.04 and P = 0.048 respectively; Table 1), resulting in a significantly lower albumin:globulin (A:G) ratio in Group A (P = 0.04; Table 1).

### 3.2 Treatment duration and efficacy

All dogs were re-examined according to the protocol. There were no statistically significant differences in the actual timing of follow-up visits between the two groups at any time point (P > 0.05; Supplementary Table 1), confirming the temporal consistency of the monitoring schedule throughout the observation period.

**Fig. 1:**
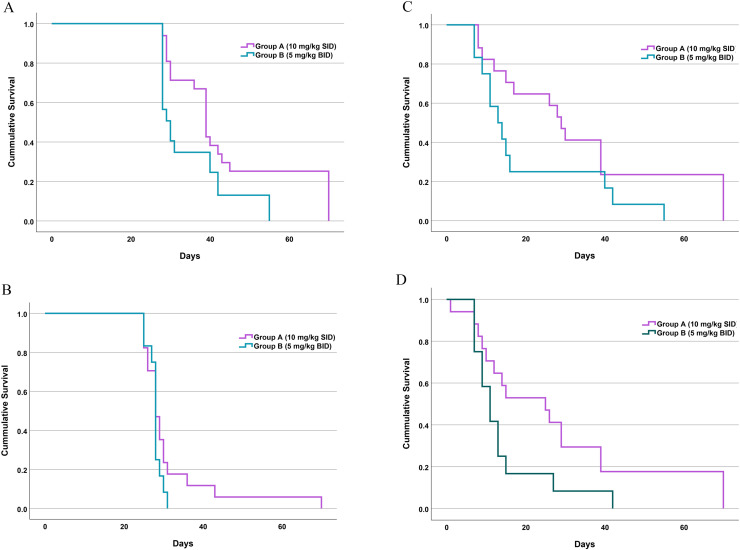
Kaplan-Meier survival analysis illustrating the comparative therapeutic efficacy and recovery kinetics between dogs treated with doxycycline 10 mg/kg SID (Group A, purple line) and 5 mg/kg BID (Group B, green line). (A) Time to treatment cessation (duration of treatment), showing a significantly shorter course of therapy in Group B (P < 0.001). (B) Time to the first negative PCR result (molecular clearance). (C) Time to quantitative platelet recovery (> 200 × 10^3^/µL). (D) Time to the observation of adequate platelet estimation on blood smears. • The cumulative probability of achieving each endpoint is plotted against time (days). Statistical significance was determined using the Log-rank test. • Abbreviations: SID, once daily; BID, twice daily.

Kaplan-Meier survival analysis revealed a significant difference in the total duration of treatment (Fig. 1A). Dogs in Group B achieved the therapeutic endpoints significantly faster than those in Group A (Log-rank P < 0.001). This is reflected in the significantly shorter treatment duration for Group B compared to Group A (P = 0.02). Dogs in Group A required a longer treatment course with a median of 39.0 days (IQR 30.0, 45.0) and a range of 28 to 70 days. Within this group, achieving the first negative PCR result took 25 - 70 days, quantitative platelet recovery took 8 - 70 days, and adequate platelet estimation took 7 - 70 days. In contrast, dogs in Group B achieved the cessation criteria significantly earlier, with a median duration of 28.5 days (IQR: 28.0 – 33.3) and a narrower range of 28 to 55 days. Correspondingly, the time to achieve the first negative PCR result was 25 - 31 days, the time to quantitative platelet recovery was 7 - 55 days, and the time to adequate platelet estimation was 7 - 42 days. Although the comparisons for these individual time-to-event endpoints molecular clearance (Log-rank P = 0.24, Fig. 1B), quantitative platelet recovery (Log-rank P = 0.08; Fig. 1C), and adequate platelet estimation (Log-rank P = 0.06; Fig. 1D) did not reach statistical significance, the descriptive ranges suggest a trend of delayed hematological recovery in a subset of Group A dogs, which ultimately contributed to their longer overall treatment course.

Regarding molecular efficacy, both regimens demonstrated high effectiveness. By Day 28, 100% of dogs in both groups tested negative for *E. canis* DNA by multiplex PCR. The median time to the first negative PCR result was comparable between Group A and Group B (28 days vs. 28 days; Log-rank P = 0.24, Fig. 1B). This negative status was sustained in all dogs tested at Day 42 and Day 70. However, at Day 98, recurrence of *E. canis* DNA was detected in two dogs (one from each group). Specifically, the dog in Group A exhibited severe thrombocytopenia (37 × 10^3^/µL) 63 days after treatment cessation, while the dog in Group B presented with mild thrombocytopenia (167 × 10^3^/µL) 70 days post-treatment. Both dogs were subsequently retreated with the original doxycycline protocol for an additional 28 days, resulting in complete clinical resolution and renewed PCR negativity.

### 3.3 Platelet Recovery

**Fig. 2:**
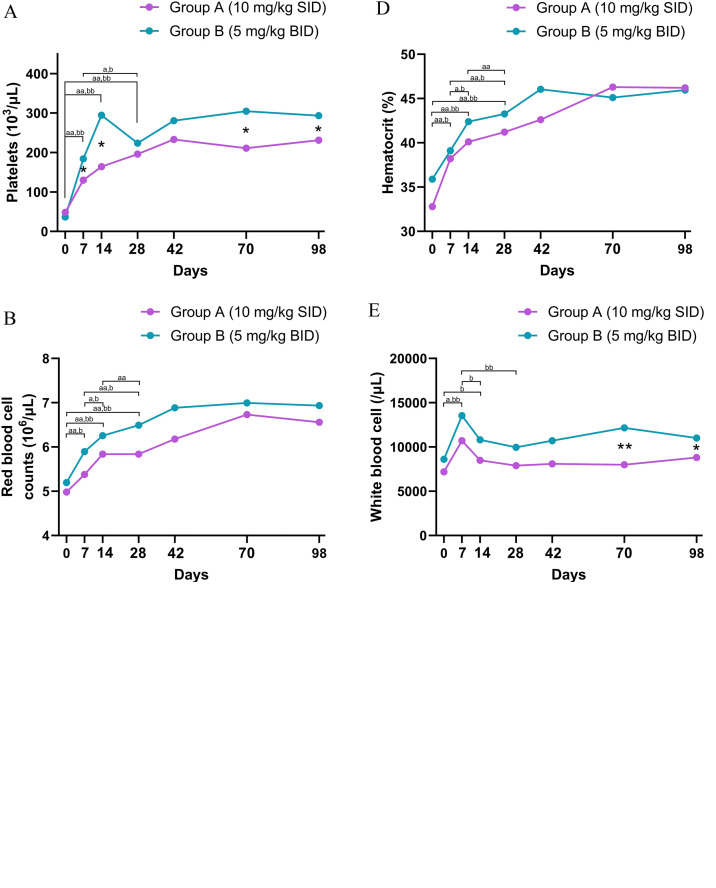

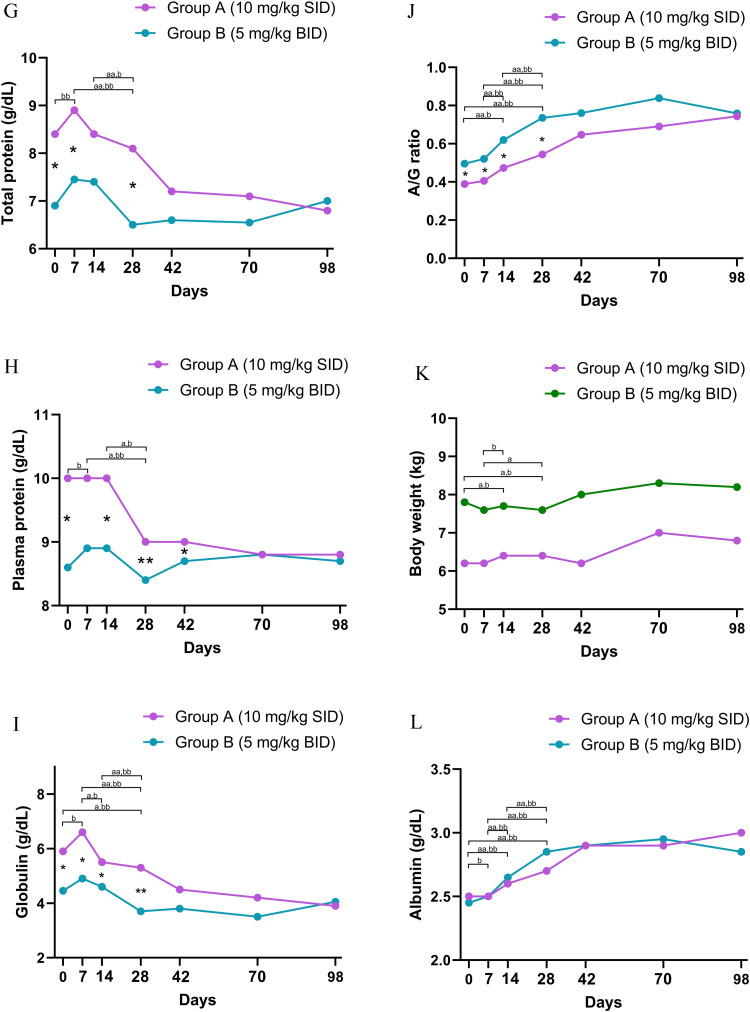
Longitudinal analysis of clinical and hematological parameters in dogs naturally infected with *Ehrlichia canis* treated with doxycycline 10 mg/kg SID (Group A; purple line and circles) and 5 mg/kg BID (Group B; green line and circles) over the 98-day study period. Data are presented as median values plotted at Days 0, 7, 14, 28, 42, 70, and 98. Statistical Significance: (1) Between-group comparison: asterrisks indicate statistically significant differences between Group A and Group B at specific time points (Mann-Whitney U test). *P < 0.05 **P < 0.01 (2) Within-group comparison: letters indicate significant differences between specific time intervals (e.g., Day 0 vs. 7, Day 0 vs. 14, etc.) within each group (Wilcoxon signed-rank test). Group A: 'a' indicates P < 0.05; 'aa' indicates P < 0.01. Group B: 'b' indicates P < 0.05; 'bb' indicates P < 0.01. Panels: (A) Platelets (B) Red blood cell counts (C) Hemoglobin (D) Hematocrit (E) White blood cell counts (F) Eosinophils (G) Total protein (H) Plasma protein (I) Globulin (J) Albumin to globulin ratio (A/G ratio) (K) Body weight (L) Albumin Abbreviations: SID, once daily; BID, twice daily.

At baseline, thrombocytopenia was a universal finding across the study population, with no statistically significant difference in initial platelet counts between the groups (P = 0.48; Table 1). While the overall time to reach the normalization threshold (> 200 × 10^3^/µL) was not statistically different between groups (Log-rank P = 0.08; Fig. 1C), analysis of platelet counts at specific time points revealed a significantly more rapid early recovery in Group B. Specifically, median platelet counts in Group B were significantly higher than Group A at Day 7 (median 184, [IQR: 165, 278] vs. 130 [IQR: 88, 178] x 10^3^/µL, P = 0.02; Fig. 2A and Supplementary Table 2) and Day 14 (median 294 [IQR: 229, 330] vs. 164 [IQR: 129, 219] x 10^3^/µL, P = 0.02; Fig. 2A and Supplementary Table 3).

By Day 28, platelet counts in 8 dogs of Group A and 10 dogs of group B had returned to within the reference range (> 200 × 10^3^/µL) with no statistic difference between the two group (Fig. 2A, Supplementary Table 4). A significant difference was observed again at Day 70 (P = 0.01) and Day 98 (P = 0.046), with Group B maintaining higher counts (Fig. 2A, Supplementary Tables 5-7).

Assessment of platelet estimation from blood smears corroborated these findings. The median time to achieve adequate platelet estimation was notably shorter in Group B (11.0 days [IQR: 7.5, 14.5) compared to Group A (25 days [IQR: 9.5, 39.0]), but not significant (Log-rank P = 0.06; Fig. 1D)

At Day 14, the proportion of dogs with adequate platelet estimation was significantly higher in Group B (91.7%) compared to Group A (52.9%) (P = 0.026).

### 3.4 Comparative analysis of hematological and biochemical parameters

#### 3.4.1 Erythrogram

Both groups showed significant improvements in red blood cell (RBC) counts, hemoglobin, and hematocrit over the first 28 days (P < 0.01; (Fig.s 2B, 2C, and 2D respectively and Supplementary Tables 8 - 9), with no significant differences between groups (P > 0.05) (Fig.s 2B, 2C, and 2D respectively and Supplementary Tables 2-7).

#### 3.4.2 Leukogram

Group B showed significantly higher white blood cell (WBC) counts than Group A at Day 70 (P < 0.001; Fig. 2E and Supplementary Table 6) and Day 98 (P = 0.027; Fig. 2E and Supplementary Table 7). Additionally, Group B exhibited significantly higher eosinophil counts at Day 7, 42, and 70 (P < 0.05; Fig. 2F and Supplementary Tables 2, 4, 6 respectively).

#### 3.4.3 Biochemistry

Significant disparities in serum protein profiles were observed between the two groups throughout the study period. Group A exhibited significantly higher concentrations of total protein, plasma protein, and globulin at baseline compared to Group B (P < 0.05; Fig. 2G, 2H, and 2I; Table 1). This pattern of elevated protein indices in Group A persisted during the treatment phase, with significantly higher levels of total protein and globulin observed at Day 7 (Fig. 2G and 2I; Supplementary Table 2), plasma protein and globulin at Day 14 (Fig. 2H and 2I; Supplementary Table 3), and all three parameters (total protein, plasma protein, and globulin) at Day 28 (Fig. 2G, 2H, and 2I; Supplementary Table 4). By Day 42, only plasma protein remained significantly higher in Group A (Fig. 2H; Supplementary Table 5). Conversely, reflecting the lower globulin burden, Group B maintained a significantly higher albumin:globulin (A:G) ratio starting from baseline (Day 0) and continuing consistently through Days 7, 14, and 28 (P < 0.05; Fig. 2J; Table 1; Supplementary Tables 2–4).

### 3.5 Longitudinal analysis of clinical and hematological parameters (Day 0 - 28)

Longitudinal monitoring from Day 0 to Day 28 showed significant recovery trends across multiple parameters. Both groups demonstrated a significant increase in body weight (P = 0.002 for Group A; P = 0.042 for Group B; Fig. 2K, Supplementary Tables 8 and 9). This physical improvement was accompanied by a marked normalization of the total protein (P = 0.001 for Group A; P < 0.001 for Group B; Fig. 2G, Supplementary Tables 8 and 9). Specifically, this was characterized by increases in albumin (Fig. 1L) concurrent with decrease in globulin levels (Fig. 1I), leading to a significantly improved A:G ratio by Day 28 (Fig. 2J; Supplementary Tables 8 and 9).

Regarding the leukocyte response, the groups exhibited distinct patterns; Group B showed a significant change in total WBC counts over time (P = 0.001) with a characteristic peak at Day 7, whereas Group A displayed no statistically significant trend in total WBC (P = 0.097) (Fig. 2E; Supplementary Tables 8 and 9).

### 3.6 Evaluation of sustained efficacy and long-term stability

To evaluate the durability of the therapeutic response, all dogs were subjected to a post-treatment monitoring period of at least 8 weeks following the cessation of doxycycline. The median duration of this drug-free follow-up phase was significantly longer in Group B (median: 70 days [IQR: 68, 73]; range: 56 - 85) compared to Group A (median: 63 days [IQR: 56, 70]; range: 56 - 91) (P = 0.036). This disparity was directly attributable to the significantly shorter treatment duration achieved in Group B, which consequently allowed for a more prolonged observation period within the study's timeframe.

#### 3.6.1 Sustained clinical improvement

**Fig. 3:**
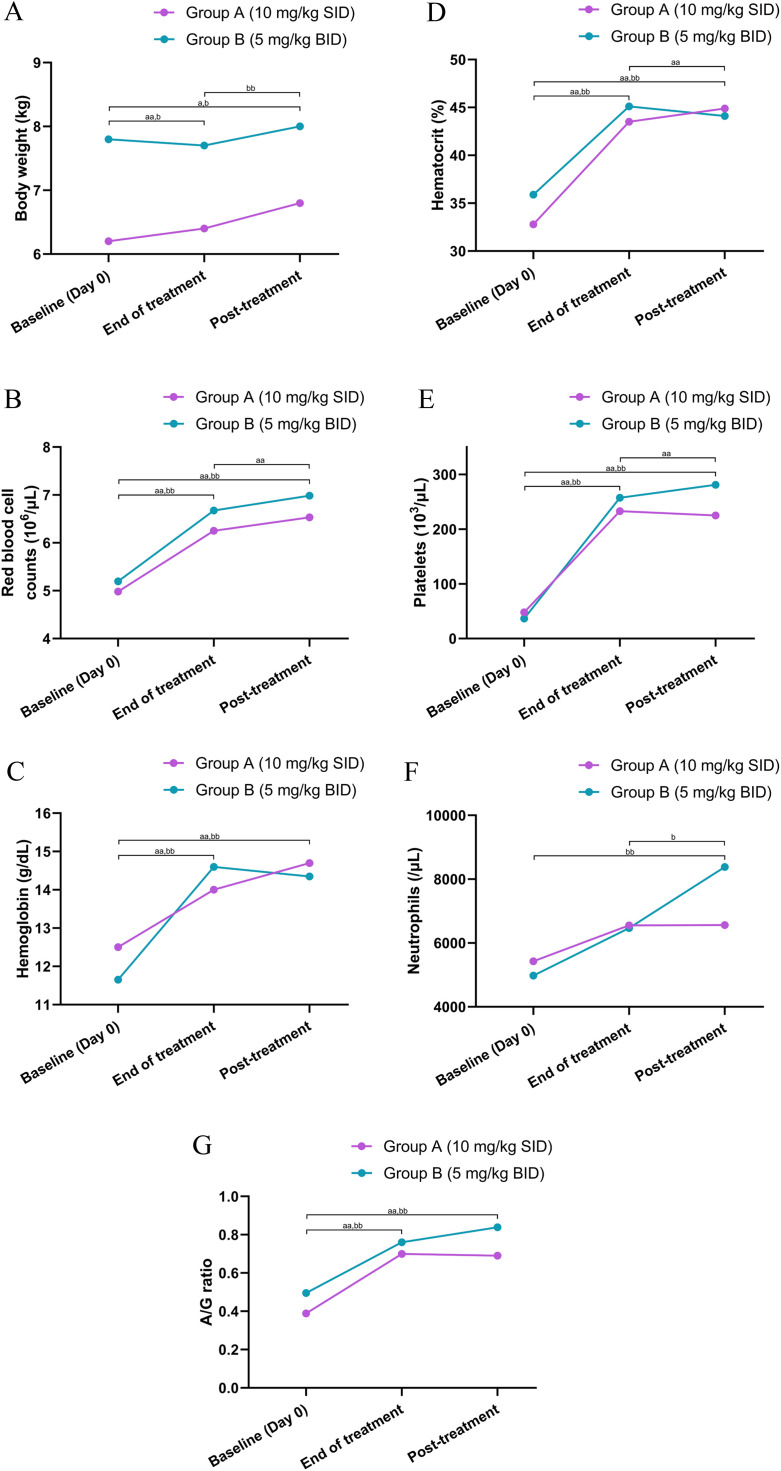
Comparative analysis of clinical and hematological parameters in dogs naturally infected with *Ehrlichia ca nis* at three key milestones: baseline (Day 0), end of treatment, and post-treatment (drug-free follow-up period). Dogs were treated with doxycycline 10 mg/kg SID (Group A; purple line and circles) or 5 mg/kg BID (Group B; green line and circles). Data are presented as median values plotted at each time point. Statistical Significance: (1) Between-group comparison: asterisks indicate statistically significant differences between Group A and Group B at specific time points (Mann-Whitney U test). *P < 0.05 **P < 0.01 (2) Within-group comparison: letters indicate significant differences between time points (Baseline vs. End of treatment, Baseline vs. Post-treatment, and End of treatment vs. Post-treatment) within each group (Wilcoxon signed-rank test). Group A: 'a' indicates P < 0.05; 'aa' indicates P < 0.01. Group B: 'b' indicates P < 0.05; 'bb' indicates P < 0.01. Panels: (A) Body weight (B) Red blood cell counts (C) Hemoglobin (D) Hematocrit (E) Platelets (F) Neutrophils (G) Albumin to globulin ratio (A/G ratio) Abbreviations: SID, once daily; BID, twice daily.

A progressive and statistically significant increase in body weight was observed in both groups (Group A: P = 0.002, Group B: P = 0.006; Fig. 3A; Supplementary Tables 10 and 11). Notably, this upward trend persisted well beyond the cessation of doxycycline therapy, indicating durable clinical recovery.

#### 3.6.2 Hematological stability

Significant improvements were observed in erythroid parameters (RBC, hemoglobin, hematocrit) and platelet counts at the end of treatment and following treatment cessation. Statistical analysis confirmed significant increases in Group A (P < 0.001, P = 0.01, P < 0.001, and P < 0.001, respectively) and Group B (P = 0.003, P < 0.001, P = 0.002, and P = 0.002, respectively). Crucially, these parameters were maintained within physiological reference ranges during the drug-free follow-up period, confirming the absence of hematological regression or relapse of anemia and thrombocytopenia (Fig. 3B – 3E; Supplementary Tables 10 and 11). Additionally, Group B demonstrated a significant upward trend in neutrophil counts (P = 0.001; Fig. 2F; Supplementary Table 11), which stabilized at physiological levels post-treatment.

#### 3.6.3 Biohemical normalization

The normalization of serum protein profiles was sustained throughout the follow-up period. Both groups exhibited a significant and persistent decrease in globulin levels concurrently with an increase in the A:G ratio (P < 0.001; Fig. 3G, Supplementary Tables 10 &11). This sustained biochemical improvement strongly indicates the successful and permanent resolution of the chronic inflammatory state associated with *E. canis* infection.

## 4 Discussion

The present study provides a prospective, randomized clinical evaluation comparing the therapeutic efficacy and rate of hematological recovery of two standard doxycycline regimens; 10 mg/kg once daily (SID) versus 5 mg/kg twice daily (BID), for the treatment of naturally occurring CME. The primary findings indicate that while both protocols are generally effective, the BID regimen demonstrated superior pharmacodynamic performance, evidenced by significantly more rapid platelet recovery and earlier molecular clearance compared to the SID regimen.

### 4.1 Comparative efficacy and molecular clearance kinetics

In terms of initial pathogen suppression, doxycycline proved highly effective. However, a distinct difference in clearance kinetics was observed between the two protocols. The BID regimen (Group B) achieved 100% molecular clearance in peripheral blood by Day 28. In contrast, the SID regimen (Group A) exhibited delayed molecular clearance in a subset of dogs; specifically, two dogs in Group A remained PCR-positive at Day 28, requiring extended treatment durations (clearing at Days 42 and 70, respectively) to achieve negativity. This finding contrasts with the 2002 ACVIM consensus statement recommending a fixed 28-day treatment course (Neer et al., 2002). In our study, delayed molecular clearance was observed in a small subset of dogs receiving the SID protocol. While it is hypothesized that dosing frequency might influence therapeutic drug exposure, plasma doxycycline concentrations were not measured in this study. Therefore, robust conclusions regarding pharmacokinetic causality cannot be drawn. Further studies with larger sample sizes and concurrent pharmacokinetic evaluations are necessary to confirm whether these dosing regimens significantly alter drug accumulation and pathogen elimination. This aligns with previous studies where 5 mg/kg BID effectively cleared infection in acute and subclinical cases (Breitschwerdt et al., 1998; Neer et al., 1999; Shropshire et al., 2018). Conversely, our observation of delayed clearance in the SID group supports reports citing persistence of infection with the once-daily regimen, indicating that the SID protocol may carry a higher risk of incomplete clearance within the standard 4-week window (Fourie et al., 2015; Harrus et al., 1998; McClure et al., 2010).

### 4.2 The challenge of sequestration and recurrence

The interpretation of "clearance" based solely on peripheral blood PCR requires caution, even after negative results are obtained. It is well-established that *E. canis* can be sequestered in the spleen and bone marrow, evading detection in the peripheral circulation. Previous studies utilizing splenic aspirates have documented persistent infection in dogs that tested PCR-negative in blood (Harrus et al., 2004; Mylonakis et al., 2019). Consequently, the negative blood PCR results observed in our study; whether achieved early (Group B) or late (Group A), should be interpreted as the successful elimination of circulating organisms rather than definitive whole-body sterilization.

This distinction is underscored by the recurrence of *E. canis* DNA detected in two dogs (one from each group) at Day 98 (approximately 60 days post-treatment cessation), accompanied by renewed thrombocytopenia. Although this post-treatment recurrence was observed, the exact mechanism remains unclear. While recrudescence from tissue reservoirs is a hypothesis supported by previous literature (Mylonakis et al., 2019), our findings must be interpreted cautiously. Despite the maintenance of topical selamectin prophylaxis, reinfection via natural tick exposure cannot be completely excluded. Furthermore, the analytical sensitivity of PCR represents a potential limitation; a negative PCR result in peripheral blood indicates that bacterial DNA has fallen below the detection threshold, but it does not definitively confirm complete pathogen elimination from all tissues.

### 4.3 Platelet recovery and baseline confounders

A pivotal finding was the significantly more rapid hematological recovery observed in Group B. Dogs in this group achieved quantitative and qualitative platelet normalization earlier than those in Group A. While this supports the pharmacodynamic superiority of the BID regimen, the influence of baseline characteristics must be acknowledged (Gutiérrez et al., 2012; Maaland et al., 2013). Group A exhibited significantly higher globulin levels at enrollment. Hyperglobulinemia is a hallmark of chronic immune stimulation in CME, suggesting that dogs in Group A may have had a higher initial inflammatory burden (Codner & Farris-Smith, 1986; Harrus et al., 1999; Mylonakis & Theodorou, 2017). While all enrolled dogs were seropositive, we acknowledge that a positive rapid antibody test does not necessarily indicate a comparable stage or duration of infection, as antibodies may persist long after initial exposure. Consequently, baseline differences between the cohorts, such as the significantly higher globulin levels observed in Group A, could still represent a potential confounding factor that might have influenced the observed hematological recovery kinetics.

### 4.4 The "treat-to-target" therapeutic approach

Current guidelines typically recommend a treatment duration of 28 days (Mylonakis et al., 2019; Neer et al., 2002). However, our results argue against a "one-size-fits-all" fixed duration, as previous studies have similarly documented the persistence of infection despite adherence to standard 4-week protocols (Fourie et al., 2015; McClure et al., 2010). If therapy had been discontinued at Day 28 based solely on the calendar, the two PCR-positive dogs in Group A would have been prematurely withdrawn from treatment, likely resulting in clinical failure or rapid recrudescence. This risk is consistent with reports indicating that clinical and hematological improvement often precedes the elimination of the organism, and premature cessation can lead to relapse (Harrus et al., 2004; Iqbal & Rikihisa, 1994). By implementing a "treat-to-target" protocol; mandating both molecular clearance and platelet normalization prior to cessation, we ensured more complete clinical resolution. This supports the recommendation that treatment duration should be individualized based on sequential clinical and hematological monitoring rather than relying on a fixed time course (Mylonakis et al., 2019). However, given the limited sample size of the present study, the observation that a subset of dogs required extended therapy to achieve PCR negativity should be interpreted with caution. Rather than a definitive recommendation for a broad change in standard treatment strategies, the 'treat-to-target' approach might be viewed as a potential clinical consideration, particularly for refractory cases or when prolonged monitoring is feasible.

### 4.5 Limitations and further investigation

Several limitations of this study warrant consideration. First, the sample size (n=29) is relatively small, which may limit the generalization of findings. Second, the randomization process resulted in a baseline imbalance of inflammatory markers (globulins) between groups, complicating the direct comparison of recovery speeds. Third, efficacy was monitored via peripheral blood PCR; tissue aspiration (spleen/bone marrow) was not performed due to the client-owned nature of the animals. Relatedly, the analytical sensitivity of PCR means that a negative result in peripheral blood does not necessarily confirm complete pathogen elimination, as bacterial loads may simply fall below the detection threshold, thereby potentially overestimating true clearance rates. Fourth, in the instances of recurrent PCR positivity, our study design does not allow for a clear differentiation between true recrudescence from tissue reservoirs and possible reinfection, despite the concurrent use of ectoparasite prophylaxis. Finally, plasma doxycycline concentrations were not measured. Future research should include pharmacokinetic studies to definitively correlate drug exposure levels with clinical recovery in naturally infected dogs. Additionally, further investigation utilizing splenic aspirates or xenodiagnosis is warranted to evaluate the comparative ability of SID versus BID regimens to eradicate sequestered tissue burdens.

## 5 Conclusions

This study demonstrates that both the 10 mg/kg SID and 5 mg/kg BID doxycycline regimens are highly effective for treating canine monocytic ehrlichiosis. However, the BID regimen may offer a trend toward faster initial hematological recovery and a significantly shorter overall treatment duration in some patients. While standard fixed-duration protocols are generally successful, individualized monitoring or a “treat-to-target’ consideration may be beneficial for cases exhibiting delayed clinical or molecular responses. Future investigations incorporating larger sample sizes and pharmacokinetic tracking are required to fully elucidate optimal dosing strategies and the precise mechanisms of post-treatment recurrence.

## Ethical approval statement

The study was approved by the Mahidol University–Institutional Animal Care and Use Committee of the Faculty of Veterinary Science (No. MUVS-2016-09-40).

## CRediT authorship contribution statement

**Mookmanee Taechikantaphat:** Writing – review & editing, Writing – original draft, Visualization, Validation, Software, Resources, Methodology, Investigation, Formal analysis, Data curation, Conceptualization. **Areerat Kongchareon:** Writing – review & editing, Writing – original draft, Visualization, Validation, Methodology, Investigation, Formal analysis. **Pruksa Julapanthong:** Writing – review & editing, Writing – original draft, Visualization, Validation, Resources, Investigation, Formal analysis. **Ladawan Sariya:** Writing – review & editing, Writing – original draft, Visualization, Validation, Resources, Methodology, Investigation, Formal analysis. **Walasinee Sakcamduang:** Writing – review & editing, Writing – original draft, Visualization, Validation, Supervision, Software, Resources, Project administration, Methodology, Investigation, Funding acquisition, Formal analysis, Data curation, Conceptualization.

## Declaration of competing interest

No conflicts of interest have been declared. While this study was financially supported by Zoetis (Thailand) Ltd, Bangkok, Thailand; the funder had no involvement in the study’s design, execution, data analysis, interpretation, or the decision to submit for publication.

## Appendix Supplementary materials

Image, application 1

Image, application 2

Image, application 3

Image, application 4

Image, application 5

Image, application 6

Image, application 7

Image, application 8

Image, application 9

Image, application 10

Image, application 11
