# Supplementary material for: Comparative efficacy of once-daily versus twice-daily doxycycline regimens in dogs naturally infected with Ehrlichia canis: A randomized clinical trial
Source: Vet Anim Sci. 2026 Apr 16;32:100661. doi: 10.1016/j.vas.2026.100661 (PMC13129463; doi:10.1016/j.vas.2026.100661)
Supplement: Supplementary file 1 [file mmc1.docx]

**Supplement Table 1:** Comparison of the actual timing of scheduled follow-up visits between dogs in Group A (10 mg/kg once daily (SID)) and Group B (5 mg/kg twice daily (BID)).

| Scheduled visit | Group A (n=17)  Actual day [Median (IQR)] | Group B (n=12)  Actual day [Median (IQR)] | P-value |
| --- | --- | --- | --- |
| Visit 1 (Day 0) | 0 (0, 0) | 0 (0, 0) | 0.26 |
| Visit 2 (Day 7) | 7 (5, 8) | 7.00 (6, 7) | 0.57 |
| Visit 3 (Day 14) | 14 (12, 15) | 14 (13, 14) | 0.54 |
| Visit 4 (Day 28) | 28 (26, 29) | 28 (27, 28) | 0.65 |
| Visit 5 (Day 42) | 42 (40, 43) | 42 (41, 43) | 0.86 |
| Visit 6 (Day 70) | 70 (68, 71) | 70 (69, 70) | 0.56 |
| Visit 7 (Day 98) | 99 (98, 101) | 98 (97, 100) | 0.21 |

- Data are presented as median and interquartile range (IQR).
- P-values derived from Mann-Whitney U test comparing differences between groups at each time point.
