# Supplementary material for: Comparative efficacy of once-daily versus twice-daily doxycycline regimens in dogs naturally infected with Ehrlichia canis: A randomized clinical trial
Source: Vet Anim Sci. 2026 Apr 16;32:100661. doi: 10.1016/j.vas.2026.100661 (PMC13129463; doi:10.1016/j.vas.2026.100661)
Supplement: Supplementary file 2 [file mmc2.docx]

**Supplementary Table 2.** Comparison of clinicopathological parameters between dogs naturally infected with *E. canis* in Group A (10 mg/kg once daily (SID)) and Group B (5 mg/kg twice daily (BID)) at Day 7 (Visit 2).

| Parameters | Group A (10 mg/kg SID) (n=17) | Group B (5 mg/kg BID) (n=12) | P-value |
| --- | --- | --- | --- |
| Body Weight (kg) | 6.2 (3.4, 8.3) | 7.6 (4.2, 12.2) | 0.18 |
| Temperature | 101.8 (101.0, 102.3) | 101.2 (101.0, 101.4) | 0.26 |
| Heart rate (beats/min) | 120 (100, 124) | 120 (100, 123) | 0.75 |
| White blood cell count (/µL) | 10700 (7450, 12700) | 13550 (11050, 15175) | 0.06 |
| Neutrophil (/µL) | 6867 (4756, 8261) | 8392 (5228, 11081) | 0.45 |
| Lymphocyte (/µL) | 2685 (1239, 3504) | 3822 (2218, 5509) | 0.13 |
| Monocyte (/µL) | 174 (83, 392) | 307 (16, 651) | 0.66 |
| Eosinophil (/µL) | 214 (0, 272) | 399 (221, 1134) | 0.02 |
| Band neutrophil (/µL) | 0 (0, 0) | 0 (0, 0) | 0.66 |
| Red blood cell count (10^6^/µL ) | 5.38 (4.20, 6.34) | 5.90 (5.07, 6.23) | 0.63 |
| Hemoglobin (g/dL) | 13.0 (9.8, 15.4) | 12.6 (10.0, 14.0) | 0.54 |
| Hematocrit % | 38.2 (29.6, 43.3) | 39.1 (33.5, 43.7) | 0.76 |
| MCV (fL) | 69 (68, 71) | 70 (66, 72) | 0.76 |
| MCH (pg) | 24.1 (22.4, 25.2) | 23.4 (22.0, 24.2) | 0.18 |
| MCHC (g/dL) | 33.9 (33.0, 35.2) | 33.1 (32.2, 34.0) | 0.12 |
| RDW (%) | 15.9 (14.6, 17.2) | 16.4 (15.2, 20.3) | 0.27 |
| Platelets (10^3^/µL ) | 130 (87, 181) | 184 (153, 295) | 0.02 |
| Platelet smear (decreased/adequate) | 12/5 | 5/7 | 0.14 |
| Plasma protein (g/dL) | 10.0 (9.1, 11.0) | 8.9 (8.6, 10.1) | 0.16 |
| Total protein (g/dL) | 8.9 (7.9, 10.4) | 7.4 (6.4, 8.8) | 0.049 |
| Albumin (g/dL) | 2.5 (2.0, 2.7) | 2.5 (2.4, 2.9) | 0.27 |
| Globulin (g/dL) | 6.6 (5.2, 8.0) | 4.9 (4.0, 6.0) | 0.04 |
| A/G ratio | 0.40 (0.28, 0.46) | 0.52 (0.45, 0.67) | 0.01 |
| ALP (u/L) | 117 (64, 256) | 230 (74, 458) | 0.19 |
| ALT (u/L) | 59 (35, 100) | 141 (40, 322) | 0.14 |
| BUN (mg/dL) | 22 (14, 30) | 15.5 (11.25, 33.25) | 0.40 |
| Creatinine (mg/dL) | 1.1 (1, 1.4) | 1.0 (0.9, 1.3) | 0.29 |
