# Supplementary material for: Comparative efficacy of once-daily versus twice-daily doxycycline regimens in dogs naturally infected with Ehrlichia canis: A randomized clinical trial
Source: Vet Anim Sci. 2026 Apr 16;32:100661. doi: 10.1016/j.vas.2026.100661 (PMC13129463; doi:10.1016/j.vas.2026.100661)
Supplement: Supplementary file 3 [file mmc3.docx]

**Supplementary Table 3.** Comparison of clinicopathological parameters between dogs naturally infected with *E. canis* in Group A (10 mg/kg once daily (SID)) and Group B (5 mg/kg twice daily (BID)) at Day 14 (Visit 3).

| Parameters | Group A (10 mg/kg SID) (n=17) | Group B (5 mg/kg BID) (n=12) | P-value |
| --- | --- | --- | --- |
| Body Weight (kg) | 6.4 (3.9, 7.6) | 7.7 (4.2, 12.8) | 0.18 |
| Temperature | 101.8 (101.4, 102.4) | 101.8 (101.0, 102.4) | 0.43 |
| Heart rate (beats/min) | 112 (98, 120) | 110 (100, 120) | 0.98 |
| White blood cell count (/µL) | 8500 (6650, 10600) | 10800 (9400, 13325) | 0.08 |
| Neutrophil (/µL) | 5695 (4255, 6970) | 6838 (5529, 8024) | 0.16 |
| Lymphocyte (/µL) | 2241 (1036, 3644) | 2649 (1508, 4772) | 0.22 |
| Monocyte (/µL) | 104 (73, 180) | 132 (16, 217) | 0.76 |
| Eosinophil (/µL) | 420 (106, 624) | 500 (299, 938) | 0.22 |
| Band neutrophil (/µL) | 0 (0, 104) | 0 (0, 0) | 0.24 |
| Red blood cell count (10^6^/µL ) | 5.84 (4.46, 6.44) | 6.26 (5.49, 6.96) | 0.18 |
| Hemoglobin (g/dL) | 13.8 (10.4, 15.0) | 14.8 (12.2, 16.4) | 0.32 |
| Hematocrit % | 40.1 (30.8, 44.8) | 42.4 (37.1, 48.7) | 0.33 |
| MCV (fL) | 69 (68, 72) | 69 (66, 71) | 0.58 |
| MCH (pg) | 23.2 (22.5, 24.2) | 22.6 (21.6, 24.5) | 0.41 |
| MCHC (g/dL) | 33.4 (32.7, 34.4) | 32.9 (31.6, 34.1) | 0.36 |
| RDW (%) | 15.6 (14.4, 16.8) | 17 (16.1, 18.2) | 0.03 |
| Platelets (10^3^/µL ) | 164 (126, 240) | 294 (229, 3601) | 0.02 |
| Platelet smear (decreased/adequate) | 8/9 | 1/11 | 0.03 |
| Plasma protein (g/dL) | 10.0 (9.0, 11.0) | 8.9 (8.2, 9.2) | 0.04 |
| Total protein (g/dL) | 8.4 (7.6, 9.9) | 7.4 (6.2, 8.4) | 0.08 |
| Albumin (g/dL) | 2.6 (2.4, 2.9) | 2.6 (2.5, 3.0) | 0.41 |
| Globulin (g/dL) | 5.5 (4.8, 7.2) | 4.6 (3.9, 5.5) | 0.046 |
| A/G ratio | 0.47 (0.33, 0.60) | 0.62 (0.51, 0.77) | 0.04 |
| ALP (u/L) | 124 (58, 222) | 270 (87, 437) | 0.20 |
| ALT (u/L) | 55 (34, 320) | 137 (29, 308) | 1.00 |
| BUN (mg/dL) | 23 (14, 28) | 15 (13, 21) | 0.29 |
| Creatinine (mg/dL) | 1 (0.9, 1.3) | 1 (0.8, 1.2) | 0.36 |
