# Supplementary material for: Comparative efficacy of once-daily versus twice-daily doxycycline regimens in dogs naturally infected with Ehrlichia canis: A randomized clinical trial
Source: Vet Anim Sci. 2026 Apr 16;32:100661. doi: 10.1016/j.vas.2026.100661 (PMC13129463; doi:10.1016/j.vas.2026.100661)
Supplement: Supplementary file 4 [file mmc4.docx]

**Supplementary Table 4.** Comparison of clinicopathological parameters between dogs naturally infected with *E. canis* in Group A (10 mg/kg once daily (SID)) and Group B (5 mg/kg twice daily (BID)) at Day 28 (Visit 4).

| Parameters | Group A (10 mg/kg SID) (n=17) | Group B (5 mg/kg BID) (n=12) | P-value |
| --- | --- | --- | --- |
| Body Weight (kg) | 6.4 (3.9, 7.9) | 7.6 (4.4, 13.0) | 0.21 |
| Temperature | 101.8 (100.9, 102.2) | 101.8 (101.3, 102.3) | 0.74 |
| Heart rate (beats/min) | 120 (92, 120) | 110 (100, 120) | 0.93 |
| White blood cell count (/µL) | 7900 (7250, 10150) | 9950 (7950, 11575) | 0.15 |
| Neutrophil (/µL) | 6142 (5100, 7865) | 7731 (4705, 8766) | 0.45 |
| Lymphocyte (/µL) | 1560 (855, 1958) | 2168 (982, 3130) | 0.13 |
| Monocyte (/µL) | 131 (69, 175) | 202 (106, 253) | 0.08 |
| Eosinophil (/µL) | 262 (84, 411) | 510 (203, 1070) | 0.11 |
| Band neutrophil (/µL) | 0 (0, 0) | 0 (0, 0) | 0.71 |
| Red blood cell count (10^6^/µL ) | 5.84 (5.04, 7.12) | 6.49 (5.92, 7.12) | 0.35 |
| Hemoglobin (g/dL) | 14.0 (11.4, 15.5) | 14.2 (12.4, 16.2) | 0.54 |
| Hematocrit % | 41.2 (33.6, 48.9) | 43.2 (38.2, 49.2) | 0.39 |
| MCV (fL) | 69 (67, 70) | 68 (64, 71) | 0.79 |
| MCH (pg) | 23.1 (22.2, 24.2) | 22.5 (21.0, 23.7) | 0.31 |
| MCHC (g/dL) | 32.8 (32.4, 34.0) | 32.7 (32.1, 33.3) | 0.52 |
| RDW (%) | 15.2 (14.7, 16.2) | 16.0 (14.9, 18.0) | 0.18 |
| Platelets (10^3^/µL ) | 196 (146, 260) | 224 (193, 307) | 0.16 |
| Platelet smear (decreased/adequate) | 5/12 | 1/11 | 0.17 |
| Plasma protein (g/dL) | 9.0 (9.0, 10.3) | 8.4 (8.0, 8.8) | 0.01 |
| Total protein (g/dL) | 8.1 (7.3, 9.2) | 6.5 (6.4, 7.8) | 0.02 |
| Albumin (g/dL) | 2.7 (2.5, 3.0) | 2.8 (2.7, 3.2) | 0.22 |
| Globulin (g/dL) | 5.3 (4.4, 6.6) | 3.7 (3.3, 4.7) | 0.01 |
| A/G ratio | 0.54 (0.36, 0.68) | 0.74 (0.67, 0.89) | 0.01 |
| ALP (u/L) | 95 (70, 225) | 140 (68, 354) | 0.40 |
| ALT (u/L) | 54 (34, 95) | 52 (26, 137) | 0.95 |
| BUN (mg/dL) | 18 (12, 28) | 16 (8, 20) | 0.43 |
| Creatinine (mg/dL) | 1.1 (0.9, 1.2) | 1.0 (0.9, 1.2) | 0.96 |
