# Supplementary material for: Comparative efficacy of once-daily versus twice-daily doxycycline regimens in dogs naturally infected with Ehrlichia canis: A randomized clinical trial
Source: Vet Anim Sci. 2026 Apr 16;32:100661. doi: 10.1016/j.vas.2026.100661 (PMC13129463; doi:10.1016/j.vas.2026.100661)
Supplement: Supplementary file 5 [file mmc5.docx]

**Supplementary Table 5.** Comparison of clinicopathological parameters between dogs naturally infected with *E. canis* in Group A (10 mg/kg once daily (SID)) and Group B (5 mg/kg twice daily (BID)) at Day 42 (Visit 5).

| Parameters | Group A (10 mg/kg SID) (n=17) | Group B (5 mg/kg BID) (n=12) | p-value |
| --- | --- | --- | --- |
| Body Weight (kg) | 6.2 (4.4, 7.8) | 8 (4.8, 13.1) | 0.22 |
| Temperature | 102.0 (101.0, 102.3) | 101.4 (100.8, 102.1) | 0.38 |
| Heart rate (beats/min) | 120 (100, 130) | 120 (100, 120) | 0.29 |
| White blood cell count (/µL) | 8100 (5800, 10550) | 10700 (8525, 11850) | 0.07 |
| Neutrophil (/µL) | 6156 (4485, 8518) | 6024 (5211, 7840) | 0.66 |
| Lymphocyte (/µL) | 1332 (354, 1942) | 2066 (1287, 3747) | 0.02 |
| Monocyte (/µL) | 89 (56, 256) | 151 (0, 318) | 0.84 |
| Eosinophil (/µL) | 258 (82, 438) | 783 (292, 1140) | 0.02 |
| Band neutrophil (/µL) | 0 (0, 36) | 0 (0, 70) | 0.93 |
| Red blood cell count (10^6^/µL ) | 6.18 (5.38, 7.15) | 6.88 (6.32, 7.20) | 0.08 |
| Hemoglobin (g/dL) | 14.0 (12.6, 16.0) | 14.6 (13.4, 17.2) | 0.34 |
| Hematocrit % | 42.6 (38.6, 48.6) | 46.0 (41.2, 49.8) | 0.34 |
| MCV (fL) | 69 (68, 72) | 68 (62, 72) | 0.19 |
| MCH (pg) | 22.8 (22.1, 24.0) | 22.2 (19.8, 24.0) | 0.49 |
| MCHC (g/dL) | 33.4 (32.0, 34.1) | 32.1 (31.4, 34.3) | 0.34 |
| RDW (%) | 15 (14.3, 15.9) | 16.05 (15.2, 17.5) | 0.04 |
| Platelets (10^3^/µL ) | 233 (192, 313) | 280 (250, 298) | 0.55 |
| Platelet smear (decreased/adequate) | 2/15 | 1/11 | 0.76 |
| Plasma protein (g/dL) | 9.0 (8.9, 10.0) | 8.7 (8.1, 9.0) | 0.01 |
| Total protein (g/dL) | 7.2 (6.8, 8.4) | 6.6 (6.2, 8.2) | 0.20 |
| Albumin (g/dL) | 2.9 (2.6, 3.0) | 2.9 (2.6, 3.2) | 0.38 |
| Globulin (g/dL) | 4.5 (3.8, 5.3) | 3.8 (3.5, 4.6) | 0.17 |
| A/G ratio | 0.65 (0.47, 0.79) | 0.76 (0.56, 0.87) | 0.19 |
| ALP (u/L) | 81 (40, 204) | 112 (61, 256) | 0.60 |
| ALT (u/L) | 46 (36, 80) | 38 (23, 126) | 0.57 |
| BUN (mg/dL) | 16 (14, 22) | 17 (12, 20) | 0.71 |
| Creatinine (mg/dL) | 1.2 (1.0, 1.4) | 1.0 (0.9, 1.2) | 0.28 |
