# Supplementary material for: Comparative efficacy of once-daily versus twice-daily doxycycline regimens in dogs naturally infected with Ehrlichia canis: A randomized clinical trial
Source: Vet Anim Sci. 2026 Apr 16;32:100661. doi: 10.1016/j.vas.2026.100661 (PMC13129463; doi:10.1016/j.vas.2026.100661)
Supplement: Supplementary file 6 [file mmc6.docx]

**Supplementary Table 6.** Comparison of clinicopathological parameters between dogs naturally infected with *E. canis* in Group A (10 mg/kg once daily (SID)) and Group B (5 mg/kg twice daily (BID)) at Day 70 (Visit 6).

| Parameters | Group A (10 mg/kg SID) (n=17) | Group B (5 mg/kg BID) (n=12) | P-value |
| --- | --- | --- | --- |
| Body Weight (kg) | 7.0 (4.6, 8.2) | 8.3 (5.2, 14.2) | 0.22 |
| Temperature | 101.8 (101.1, 102.2) | 101.0 (100.6, 101.8) | 0.15 |
| Heart rate (beats/min) | 120 (100, 120) | 120 (100, 120) | 0.92 |
| White blood cell count (/µL) | 8000 (6810, 10350) | 12150 (10125, 13950) | <0.001 |
| Neutrophil (/µL) | 6177 (5176, 7564) | 7795 (6205, 10583) | 0.03 |
| Lymphocyte (/µL) | 1316 (829, 1772) | 2062 (1184, 3915) | 0.07 |
| Monocyte (/µL) | 112 (58, 305) | 123 (17, 371) | 0.95 |
| Eosinophil (/µL) | 290 (82, 408) | 487 (320, 1267) | 0.01 |
| Band neutrophil (/µL) | 0 (0, 0) | 0 (0, 81) | 0.18 |
| Red blood cell count (10^6^/µL ) | 6.73 (5.78, 7.48) | 7.00 (6.24, 7.38) | 0.55 |
| Hemoglobin (g/dL) | 15.1 (13.6, 16.8) | 15.0 (13.7, 17.5) | 0.97 |
| Hematocrit % | 46.3 (41, 51.1) | 45.1 (42.6, 50.4) | 0.81 |
| MCV (fL) | 69 (67, 72) | 68 (63, 70) | 0.35 |
| MCH (pg) | 22.4 (21.8, 23.8) | 23.1 (20.4, 24.1) | 1.00 |
| MCHC (g/dL) | 32.9 (32.0, 33.4) | 33.0 (32.3, 34.4) | 0.31 |
| RDW (%) | 15.1 (14.0, 15.8) | 16.2 (15.2, 17.6) | 0.16 |
| Platelets (10^3^/µL ) | 211 (200, 276) | 304 (276, 338) | 0.01 |
| Platelet smear (decreased/adequate) | 1/16 | 0/12 | 0.39 |
| Plasma protein (g/dL) | 8.8 (8.0, 9.2) | 8.8 (8.1, 9.2) | 0.69 |
| Total protein (g/dL) | 7.1 (6.6, 8.0) | 6.6 (5.9, 8.1) | 0.30 |
| Albumin (g/dL) | 2.9 (2.4, 3.1) | 3.0 (2.7, 3.3) | 0.40 |
| Globulin (g/dL) | 4.2 (3.6, 5.2) | 3.5 (3.2, 4.6) | 0.16 |
| A/G ratio | 0.69 (0.49, 0.83) | 0.84 (0.73, 0.98) | 0.08 |
| ALP (u/L) | 60 (40, 186) | 98 (56, 156) | 0.76 |
| ALT (u/L) | 33 (28, 73) | 30 (24, 57) | 0.63 |
| BUN (mg/dL) | 15 (10, 22) | 18 (14, 22) | 0.30 |
| Creatinine (mg/dL) | 1.1 (1.0, 1.2) | 1.1 (0.9, 1.2) | 0.86 |
