# Supplementary material for: Comparative efficacy of once-daily versus twice-daily doxycycline regimens in dogs naturally infected with Ehrlichia canis: A randomized clinical trial
Source: Vet Anim Sci. 2026 Apr 16;32:100661. doi: 10.1016/j.vas.2026.100661 (PMC13129463; doi:10.1016/j.vas.2026.100661)
Supplement: Supplementary file 7 [file mmc7.docx]

**Supplementary Table 7.** Comparison of clinicopathological parameters between dogs naturally infected with *E. canis* in Group A (10 mg/kg once daily (SID)) and Group B (5 mg/kg twice daily (BID)) at Day 98 (Visit 7).

| Parameters | Group A (10 mg/kg SID) (n=17) | Group B (5 mg/kg BID) (n=12) | P-value |
| --- | --- | --- | --- |
| Body Weight (kg) | 6.8 (4.7, 9.4) | 8.2 (5.4, 13.8) | 0.22 |
| Temperature | 101.5 (100.8, 101.8) | 101.2 (101, 101.6) | 0.93 |
| Heart rate (beats/min) | 120 (100, 120) | 120 (103, 120) | 1.00 |
| White blood cell count (/µL) | 8800 (6550, 9740) | 11000 (7925, 12425) | 0.03 |
| Neutrophil (/µL) | 6624 (5078, 7595) | 7851 (5615, 9853) | 0.10 |
| Lymphocyte (/µL) | 1196 (784, 1820) | 1668 (1528, 2446) | 0.03 |
| Monocyte (/µL) | 97 (0, 218) | 127 (76, 621) | 0.20 |
| Eosinophil (/µL) | 315 (143, 684) | 364 (176, 468) | 0.93 |
| Band neutrophil (/µL) | 0 (0, 0) | 0 (0, 0) | 0.42 |
| Red blood cell count (10^6^/µL ) | 6.56 (5.82, 7.36) | 6.94 (6.41, 7.28) | 0.30 |
| Hemoglobin (g/dL) | 15.3 (13.6, 16.4) | 15.4 (14.8, 16.8) | 0.55 |
| Hematocrit % | 46.2 (40.2, 49.2) | 46.0 (45.1, 49.0) | 0.74 |
| MCV (fL) | 68 (67, 72) | 67 (63, 70) | 0.28 |
| MCH (pg) | 22.9 (22.2, 24.0) | 23.0 (20.9, 24.2) | 0.95 |
| MCHC (g/dL) | 33.0 (32.8, 34.1) | 34.0 (32.6, 35.1) | 0.54 |
| RDW (%) | 14.5 (13.6, 16.4) | 15.5 (14.5, 16.9) | 0.38 |
| Platelets (10^3^/µL ) | 231 (190, 263) | 294 (226, 335) | 0.046 |
| Platelet smear (decreased/adequate) | 3/14 | 1/11 | 0.47 |
| Plasma protein (g/dL) | 8.8 (8.2, 9.4) | 8.7 (8.0, 9.2) | 0.46 |
| Total protein (g/dL) | 6.8 (6.2, 7.7) | 7 (6.3, 8.0) | 0.89 |
| Albumin (g/dL) | 3.0 (2.7, 3.1) | 2.8 (2.7, 3.3) | 0.95 |
| Globulin (g/dL) | 3.9 (3.4, 4.8) | 4.0 (3.4, 4.8) | 0.81 |
| A/G ratio | 0.74 (0.56, 0.92) | 0.76 (0.55, 0.85) | 1.00 |
| ALP (u/L) | 57 (34, 174) | 68 (45, 136) | 0.89 |
| ALT (u/L) | 33 (28, 72) | 46 (28, 72) | 0.55 |
| BUN (mg/dL) | 18 (13, 24) | 15 (10, 23) | 0.76 |
| Creatinine (mg/dL) | 1.1 (0.9, 1.2) | 1.1 (1, 1.1) | 0.74 |
