# Supplementary material for: Comparative efficacy of once-daily versus twice-daily doxycycline regimens in dogs naturally infected with Ehrlichia canis: A randomized clinical trial
Source: Vet Anim Sci. 2026 Apr 16;32:100661. doi: 10.1016/j.vas.2026.100661 (PMC13129463; doi:10.1016/j.vas.2026.100661)
Supplement: Supplementary file 10 [file mmc10.docx]

**Supplementary Table 10.** Longitudinal assessment of clinical and clinicopathological parameters in dogs naturally infected with *E. canis* treated with doxycycline 10 mg/kg once daily (SID) (Group A) across baseline, end of treatment, and post-treatment follow-up time points.

| Parameters | N | Median (interquartile) | Overall P -value^a^ | | Pairwise comparisons^b^ | |
| --- | --- | --- | --- | --- | --- | --- |
|  |  |  |  |  | vs baseline | vs end of treatment |
| Body weight (kg) |  |  | | 0.002 |  |  |
| Baseline (Day 0) | 17 | 6.2 (3.2, 7.5) | |  |  |  |
| End of treatment | 17 | 6.4 (4.5, 7.9) | |  | 0.006 |  |
| Post-treatment | 17 | 6.8 (4.5, 8.2) | |  | 0.01 | 0.32 |
| White blood cell count (/µL) |  |  | | 0.66 |  |  |
| Baseline (Day 0) | 17 | 7200 (6000, 9250) | |  |  |  |
| End of treatment | 17 | 8100 (7450, 10400) | |  | 0.28 |  |
| Post-treatment | 17 | 8200 (6350, 9800) | |  | 0.76 | 0.29 |
| Neutrophil (/µL) |  |  | | 0.59 |  |  |
| Baseline (Day 0) | 17 | 5428 (4722, 6355) | |  |  |  |
| End of treatment | 17 | 6552 (5251, 7978) | |  | 0.18 |  |
| Post-treatment | 17 | 6560 (5094, 8336) | |  | 0.38 | 0.46 |
| Lymphocyte (/µL) |  |  | | 0.33 |  |  |
| Baseline (Day 0) | 17 | 1116 (762, 2622) | |  |  |  |
| End of treatment | 17 | 1376 (794, 2048) | |  | 0.41 |  |
| Post-treatment | 17 | 1196 (677, 1588) | |  | 0.10 | 0.06 |
| Monocyte (/µL) |  |  | | 0.08 |  |  |
| Baseline (Day 0) | 17 | 162 (96, 279) | |  |  |  |
| End of treatment | 17 | 87 (20, 288) | |  | 0.50 |  |
| Post-treatment | 17 | 110 (75, 305) | |  | 0.91 | 0.38 |
| Eosinophil (/µL) |  |  | | 0.009 |  |  |
| Baseline (Day 0) | 17 | 54 (0, 176) | |  |  |  |
| End of treatment | 17 | 300 (139, 413) | |  | 0.01 |  |
| Post-treatment | 17 | 290 (100, 570) | |  | 0.002 | 0.71 |
| Red blood cell count (10^6^/µL ) |  |  | | <0.001 |  |  |
| Baseline (Day 0) | 17 | 4.98 (3.54, 6.17) | |  |  |  |
| End of treatment | 17 | 6.25 (5.44, 7.38) | |  | <0.001 |  |
| Post-treatment | 17 | 6.53 (5.5, 7.36) | |  | <0.001 | 0.008 |
| Hemoglobin (g/dL) |  |  | | 0.01 |  |  |
| Baseline (Day 0) | 17 | 12.5 (8.3, 15.65) | |  |  |  |
| End of treatment | 17 | 14 (12.6, 16.45) | |  | 0.005 |  |
| Post-treatment | 17 | 14.7 (13, 16.75) | |  | 0.002 | 0.11 |
| Hematocrit % |  |  | | <0.001 |  |  |
| Baseline (Day 0) | 17 | 32.8 (24.6, 43.5) | |  |  |  |
| End of treatment | 17 | 43.5 (38.7, 51.0) | |  | <0.001 |  |
| Post-treatment | 17 | 44.9 (38.2, 51.4) | |  | <0.001 | 0.14 |
| MCV (fL) |  |  | | 0.08 |  |  |
| Baseline (Day 0) | 17 | 70 (67, 72) | |  |  |  |
| End of treatment | 17 | 70 (68, 72) | |  | 0.25 |  |
| Post-treatment | 17 | 69 (67, 72) | |  | 0.89 | 0.01 |
| MCH (pg) |  |  | | 0.06 |  |  |
| Baseline (Day 0) | 17 | 24.5 (23.3, 26.2) | |  |  |  |
| End of treatment | 17 | 23.0 (22.1, 24.3) | |  | 0.044 |  |
| Post-treatment | 17 | 22.7 (22.0, 24.0) | |  | 0.009 | 0.30 |
| MCHC (g/dL) |  |  | | 0.005 |  |  |
| Baseline (Day 0) | 17 | 35.3 (33.85, 37.1) | |  |  |  |
| End of treatment | 17 | 33.0 (32.0, 33.8) | |  | 0.003 |  |
| Post-treatment | 17 | 32.9 (31.9, 33.8) | |  | 0.004 | 1 |
| RDW (%) |  |  | | 0.027 |  |  |
| Baseline (Day 0) | 17 | 15.6 (14.6, 17.2) | |  |  |  |
| End of treatment | 17 | 15.2 (14.3, 16.2) | |  | 0.05 |  |
| Post-treatment | 17 | 14.9 (13.8, 16.0) | |  | 0.03 | 0.2 |
| Platelets (10^3^/µL ) |  |  | | <0.001 |  |  |
| Baseline (Day 0) | 17 | 48 (26, 112) | |  |  |  |
| End of treatment | 17 | 233 (194, 311) | |  | <0.001 |  |
| Post-treatment | 17 | 225 (190, 254) | |  | <0.001 | 0.16 |
| Plasma protein (g/dL) |  |  | | 0.049 |  |  |
| Baseline (Day 0) | 17 | 10.0 (8.3, 11.6) | |  |  |  |
| End of treatment | 17 | 9.0 (8.7, 10.3) | |  | 0.14 |  |
| Post-treatment | 17 | 9.0 (8.2, 9.4) | |  | 0.03 | 0.14 |
| Total protein (g/dL) |  |  | |  |  |  |
| Baseline (Day 0) | 17 | 8.4 (7.2, 10.4) | | <0.001 |  |  |
| End of treatment | 17 | 7.3 (6.8, 8.2) | |  | 0.005 |  |
| Post-treatment | 17 | 6.9 (6.4, 8.0) | |  | 0.002 | 0.03 |
| Albumin (g/dL) |  |  | |  |  |  |
| Baseline (Day 0) | 17 | 2.5 (1.9, 2.7) | | <0.001 |  |  |
| End of treatment | 17 | 3.0 (2.6, 3.1) | |  | <0.001 |  |
| Post-treatment | 17 | 2.9 (2.5, 3.1) | |  | 0.001 | 0.83 |
| Globulin (g/dL) |  |  | |  |  |  |
| Baseline (Day 0) | 17 | 5.8 (4.7, 8.0) | | <0.001 |  |  |
| End of treatment | 17 | 4.4 (3.8, 5.2) | |  | 0.001 |  |
| Post-treatment | 17 | 4.0 (3.4, 4.8) | |  | <0.001 | 0.06 |
| A/G ratio |  |  | |  |  |  |
| Baseline (Day 0) | 17 | 0.39 (0.26, 0.51) | | <0.001 |  |  |
| End of treatment | 17 | 0.70 (0.49, 0.80) | |  | <0.001 |  |
| Post-treatment | 17 | 0.69 (0.55, 0.86) | |  | <0.001 | 0.20 |
| ALP (u/L) |  |  | | 0.29 |  |  |
| Baseline (Day 0) | 17 | 125 (52, 174) | |  |  |  |
| End of treatment | 17 | 80 (43, 232) | |  | 0.65 |  |
| Post-treatment | 17 | 81 (42, 184) | |  | 0.39 | 0.02 |
| ALT (u/L) |  |  | | 0.003 |  |  |
| Baseline (Day 0) | 17 | 45 (30, 72) | |  |  |  |
| End of treatment | 17 | 53 (38, 90) | |  | 0.37 |  |
| Post-treatment | 17 | 31 (30, 38) | |  | 0.06 | 0.001 |
| BUN (mg/dL) |  |  | | 0.02 |  |  |
| Baseline (Day 0) | 17 | 23 (12, 32) | |  |  |  |
| End of treatment | 17 | 20 (14, 27) | |  | 0.19 |  |
| Post-treatment | 17 | 16 (11, 24) | |  | 0.01 | 0.07 |
| Creatinine (mg/dL) |  |  | | 0.26 |  |  |
| Baseline (Day 0) | 17 | 1.0 (0.8, 1.4) | |  |  |  |
| End of treatment | 17 | 1.1 (1.0, 1.4) | |  | 0.22 |  |
| Post-treatment | 17 | 1.1 (1.0, 1.4) | |  | 0.26 | 0.42 |

- Data are presented as median (interquartile range).
- ^a^P-value derived from Friedman test indicating overall difference across the three time points.
- ^b^P-values derived from Wilcoxon signed-rank test for pairwise comparisons.
- End of treatment: end of treatment visit (day of treatment cessation based on protocol criteria).
- Post-treatment: post-treatment follow-up visit (drug-free period > 8 weeks).
- *Statistically significant difference (P < 0.05).
