# Supplementary material for: Comparative efficacy of once-daily versus twice-daily doxycycline regimens in dogs naturally infected with Ehrlichia canis: A randomized clinical trial
Source: Vet Anim Sci. 2026 Apr 16;32:100661. doi: 10.1016/j.vas.2026.100661 (PMC13129463; doi:10.1016/j.vas.2026.100661)
Supplement: Supplementary file 11 [file mmc11.docx]

**Supplementary Table 11.** Longitudinal assessment of clinical and clinicopathological parameters in dogs naturally infected with *E. canis* treated with doxycycline 5 mg/kg twice daily (BID) (Group B) across baseline, end of treatment, and post-treatment follow-up time points.

| Parameters | N | Median (interquartile) | Overall P-value^a^ | Pairwise comparisons^b^ | |
| --- | --- | --- | --- | --- | --- |
|  |  |  |  | vs baseline | vs end of treatment |
| Body weight (kg) |  |  | 0.006 |  |  |
| Baseline (Day 0) | 12 | 7.8 (3.8, 12.0) |  |  |  |
| End of treatment | 12 | 7.7 (4.6, 13.1) |  | 0.03 |  |
| Post-treatment | 12 | 8 (4.9, 14.2) |  | 0.01 | 0.009 |
| White blood cell count (/µL) |  |  | 0.001 |  |  |
| Baseline (Day 0) | 12 | 8600 (5665, 10025) |  |  |  |
| End of treatment | 12 | 9600 (8525, 11650) |  | 0.18 |  |
| Post-treatment | 12 | 12500 (10200, 14475) |  | 0.002 | 0.01 |
| Neutrophil (/µL) |  |  | 0.001 |  |  |
| Baseline (Day 0) | 12 | 4976 (3766, 7483) |  |  |  |
| End of treatment | 12 | 6466 (5211, 8536) |  | 0.07 |  |
| Post-treatment | 12 | 8382 (6205, 10583) |  | 0.006 | 0.01 |
| Lymphocyte (/µL) |  |  | 0.17 |  |  |
| Baseline (Day 0) | 12 | 1818 (959, 2902) |  |  |  |
| End of treatment | 12 | 1898 (1135, 3150) |  | 1.00 |  |
| Post-treatment | 12 | 2415 (1184, 3884) |  | 0.43 | 0.03 |
| Monocyte (/µL) |  |  | 0.92 |  |  |
| Baseline (Day 0) | 12 | 290 (170, 434) |  |  |  |
| End of treatment | 12 | 171 (0, 373) |  | 0.18 |  |
| Post-treatment | 12 | 131 (18, 444) |  | 0.48 | 0.75 |
| Eosinophil (/µL) |  |  | 0.001 |  |  |
| Baseline (Day 0) | 12 | 37 (0, 139) |  |  |  |
| End of treatment | 12 | 814.5 (226, 1166) |  | 0.004 |  |
| Post-treatment | 12 | 436 (278, 1267) |  | 0.003 | 0.48 |
| Red blood cell count (10^6^/µL ) |  |  | 0.001 |  |  |
| Baseline (Day 0) | 12 | 5.20 (3.70, 5.96) |  |  |  |
| End of treatment | 12 | 6.68 (6.22, 6.91) |  | 0.003 |  |
| Post-treatment | 12 | 6.98 (6.06, 7.29) |  | 0.003 | 0.35 |
| Hemoglobin (g/dL) |  |  | <0.001 |  |  |
| Baseline (Day 0) | 12 | 11.6 (7.8, 13.3) |  |  |  |
| End of treatment | 12 | 14.6 (13.4, 15.8) |  | 0.002 |  |
| Post-treatment | 12 | 14.4 (13.7, 17.2) |  | 0.002 | 0.16 |
| Hematocrit % |  |  | 0.001 |  |  |
| Baseline (Day 0) | 12 | 35.9 (23.6, 39.1) |  |  |  |
| End of treatment | 12 | 45.1 (40.8, 49.1) |  | 0.003 |  |
| Post-treatment | 12 | 44.1 (41.3, 49.8) |  | 0.005 | 0.84 |
| MCV (fL) |  |  | 0.178 |  |  |
| Baseline (Day 0) | 12 | 68 (66, 71) |  |  |  |
| End of treatment | 12 | 67.5 (62.5, 71.0) |  | 0.50 |  |
| Post-treatment | 12 | 67.8 (63.0, 72.9) |  | 0.46 | 0.57 |
| MCH (pg) |  |  | 0.205 |  |  |
| Baseline (Day 0) | 12 | 22.3 (21.5, 23.7) |  |  |  |
| End of treatment | 12 | 22.2 (19.8, 23.7) |  | 0.62 |  |
| Post-treatment | 12 | 23.0 (20.4, 24.4) |  | 0.24 | 0.008 |
| MCHC (g/dL) |  |  | 0.016 |  |  |
| Baseline (Day 0) | 12 | 33.0 (32.0, 34.0) |  |  |  |
| End of treatment | 12 | 32.1 (31.4, 33.3) |  | 0.33 |  |
| Post-treatment | 12 | 33.0 (32.3, 34.3) |  | 0.50 | 0.004 |
| RDW (%) |  |  | 0.556 |  |  |
| Baseline (Day 0) | 12 | 15.2 (14.9, 19.1) |  |  |  |
| End of treatment | 12 | 15.8 (14.6, 17.9) |  | 0.59 |  |
| Post-treatment | 12 | 16.0 (15.2, 17.5) |  | 0.94 | 0.69 |
| Platelets (10^3^/µL ) |  |  | <0.001 |  |  |
| Baseline (Day 0) | 12 | 36 (29, 81) |  |  |  |
| End of treatment | 12 | 258(223, 298) |  | 0.002 |  |
| Post-treatment | 12 | 281 (266, 333 |  | 0.002 | 0.06 |
| Plasma protein (g/dL) |  |  | 0.794 |  |  |
| Baseline (Day 0) | 12 | 8.6 (7.8, 9.6) |  |  |  |
| End of treatment | 12 | 8.7 (8.0, 9.2) |  | 0.88 |  |
| Post-treatment | 12 | 8.7 (8.1, 9.0) |  | 0.48 | 0.31 |
| Total protein (g/dL) |  |  | 0.423 |  |  |
| Baseline (Day 0) | 12 | 6.9 (6.2, 8.6) |  |  |  |
| End of treatment | 12 | 6.6 (6.2, 7.8) |  | 0.18 |  |
| Post-treatment | 12 | 6.6 (5.9, 8.2) |  | 0.37 | 0.82 |
| Albumin (g/dL) |  |  | 0.003 |  |  |
| Baseline (Day 0) | 12 | 2.4 (2.1, 2.7) |  |  |  |
| End of treatment | 12 | 2.8 (2.6, 3.1) |  | 0.008 |  |
| Post-treatment | 12 | 3.0 (2.7, 3.4) |  | 0.003 | 0.17 |
| Globulin (g/dL) |  |  | 0.004 |  |  |
| Baseline (Day 0) | 12 | 4.4 (4.2, 5.9) |  |  |  |
| End of treatment | 12 | 3.7 (3.4, 4.7) |  | 0.003 |  |
| Post-treatment | 12 | 3.5 (3.1, 4.6) |  | 0.01 | 0.57 |
| A/G ratio |  |  | <0.001 |  |  |
| Baseline (Day 0) | 12 | 0.50 (0.43, 0.61) |  |  |  |
| End of treatment | 12 | 0.76 (0.56, 0.88) |  | 0.003 |  |
| Post-treatment | 12 | 0.84 (0.73, 0.98) |  | 0.002 | 0.18 |
| ALP (u/L) |  |  | <0.001 |  |  |
| Baseline (Day 0) | 12 | 134 (72, 235) |  |  |  |
| End of treatment | 12 | 126 (63, 277) |  | 0.27 |  |
| Post-treatment | 12 | 98 (55, 156) |  | 0.03 | 0.002 |
| ALT (u/L) |  |  | 0.116 |  |  |
| Baseline (Day 0) | 12 | 68 (36, 120) |  |  |  |
| End of treatment | 12 | 38 (27, 132) |  | 0.50 |  |
| Post-treatment | 12 | 30 (22, 79) |  | 0.03 | 0.02 |
| BUN (mg/dL) |  |  | 0.754 |  |  |
| Baseline (Day 0) | 12 | 14 (12, 26) |  |  |  |
| End of treatment | 12 | 16 (11, 19) |  | 0.53 |  |
| Post-treatment | 12 | 18 (16, 19) |  | 0.82 | 0.97 |
| Creatinine (mg/dL) |  |  | 0.105 |  |  |
| Baseline (Day 0) | 12 | 1.0 (0.8, 1.1) |  |  |  |
| End of treatment | 12 | 1.0 (0.9, 1.2) |  | 0.04 |  |
| Post-treatment | 12 | 1.0 (0.9, 1.2) |  | 0.04 | 0.80 |

- Data are presented as median (interquartile range).
- ^a^P-value derived from Friedman test indicating overall difference across the three time points.
- ^b^P-values derived from Wilcoxon signed-rank test for pairwise comparisons.
- End of treatment: end of treatment visit (day of treatment cessation based on protocol criteria).
- Post-treatment: post-treatment follow-up visit (drug-free period > 8 weeks).
- *Statistically significant difference (P < 0.05).
- Abbreviations: SID, once daily; BID, twice daily; MCV: mean corpuscular volume; MCH: mean corpuscular hemoglobin; MCHC: mean corpuscular hemoglobin concentration; RDW: red cell distribution width; A/G ratio, albumin to globulin ratio; ALP: alkaline phosphatase; ALT: alanine transferase; BUN: blood urea nitrogen.
